# Chronic fetal hypoxia disrupts the peri‐conceptual environment in next‐generation adult female rats

**DOI:** 10.1113/JP277431

**Published:** 2019-03-24

**Authors:** Catherine E. Aiken, Jane L. Tarry‐Adkins, Ana‐Mishel Spiroski, Anna M. Nuzzo, Thomas J. Ashmore, Alessandro Rolfo, Megan J. Sutherland, Emily J. Camm, Dino A. Giussani, Susan E. Ozanne

**Affiliations:** ^1^ University of Cambridge Metabolic Research Laboratories and MRC Metabolic Diseases Unit Wellcome Trust‐MRC Institute of Metabolic Science, Addenbrooke's Treatment Centre, Addenbrooke's Hospital Cambridge UK; ^2^ University Department of Obstetrics and Gynaecology University of Cambridge, Cambridge UK; ^3^ Department of Physiology, Development and Neuroscience University of Cambridge Cambridge UK; ^4^ Dipartimento di Scienze Chirurgiche Universita degli Studi di Torino Turin Italy

**Keywords:** Developmental programming, oviducts, hypoxia, infertility, reproductive ageing

## Abstract

**Key points:**

- Exposure to chronic hypoxia during gestation influences long‐term health and development, including reproductive capacity, across generations.
- If the peri‐conceptual environment in the developing oviduct is affected by gestational hypoxia, then this could have implications for later fertility and the health of future generations.
- In the present study, we show that the oviducts of female rats exposed to chronic hypoxia *in utero* have reduced telomere length, decreased mitochondrial DNA biogenesis and increased oxidative stress
- The results of the present study show that exposure to chronic gestational hypoxia leads to accelerated ageing of the oviduct in early adulthood and they help us understand how exposure to hypoxia during development could influence reproductive health across generations.

**Abstract:**

Exposure to chronic hypoxia during fetal development has important effects on immediate and long‐term outcomes in offspring. Adverse impacts in adult offspring include impairment of cardiovascular function, metabolic derangement and accelerated ovarian ageing. However, it is not known whether other aspects of the female reproductive system may be similarly affected. In the present study, we examined the impact of chronic gestational hypoxia on the developing oviduct. Wistar rat dams were randomized to either normoxia (21%) or hypoxia (13%) from day 6 post‐mating until delivery. Post‐delivery female offspring were maintained in normoxia until 4 months of age. Oviductal gene expression was assayed at the RNA (quantitative RT‐PCR) and protein (western blotting) levels. Oviductal telomere length was assayed using Southern blotting. Oviductal telomere length was reduced in the gestational hypoxia‐exposed animals compared to normoxic controls (*P* < 0.01). This was associated with a specific post‐transcriptional reduction in the KU70 subunit of DNA‐pk in the gestational hypoxia‐exposed group (*P* < 0.05). Gestational hypoxia‐exposed oviducts also showed evidence of decreased mitochondrial DNA biogenesis, reduced mtDNA copy number (*P* < 0.05) and reduced gene expression of *Tfam* (*P* < 0.05) and *Pgc1α* (*P* < 0.05). In the hypoxia‐exposed oviducts, there was upregulation of mitochondrial‐specific anti‐oxidant defence enzymes (MnSOD; *P* < 0.01). Exposure to chronic gestational hypoxia leads to accelerated ageing of the oviduct in adulthood. The oviduct plays a central role in early development as the site of gamete transport, syngamy, and early development; hence, accelerated ageing of the oviductal environment could have important implications for fertility and the health of future generations.

## Key points

- Exposure to chronic hypoxia during gestation influences long‐term health and development, including reproductive capacity, across generations.
- If the peri‐conceptual environment in the developing oviduct is affected by gestational hypoxia, then this could have implications for later fertility and the health of future generations.
- In the present study, we show that the oviducts of female rats exposed to chronic hypoxia *in utero* have reduced telomere length, decreased mitochondrial DNA biogenesis and increased oxidative stress
- The results of the present study show that exposure to chronic gestational hypoxia leads to accelerated ageing of the oviduct in early adulthood and they help us understand how exposure to hypoxia during development could influence reproductive health across generations.

## Introduction

Many human fetuses are exposed to chronic gestational hypoxia, either via factors intrinsic to the pregnancy, such as impaired utero–placental blood flow (Kuzmina *et al*. 2005), or via factors arising from the maternal environment, such as pregnancy at high altitude (Ducsay, 1998; Postigo *et al*. 2009; Giussani *et al*. 2016). The immediate effects of gestational hypoxia have been characterized in both human pregnancies and animal models, and include adverse outcomes such as intrauterine growth restriction, low birth weight and stillbirth (Giussani *et al*. 2001; Keyes *et al*. 2003; Richter *et al*. 2012; Gonzalez‐Candia *et al*. 2016). The long‐term outcomes for the adult offspring of chronic gestational hypoxia are generally less well understood, although some aspects, such as the increased risk of later cardiovascular dysfunction, have been well described in animal models (Giussani *et al*. 2012; Giussani & Davidge, 2013). Furthermore, there is evidence from animal models that exposure to chronic gestational hypoxia can adversely impact brain development (Phillips *et al*. 2017), renal ageing (Gonzalez‐Rodriguez *et al*. 2013) and insulin resistance (Camm *et al*. 2011).

The link between exposure to various suboptimal intrauterine environments and subsequent impairment of reproductive function has been demonstrated in a number of animal models (Aiken *et al*. 2013; Aiken *et al*. 2016). These studies have mainly been performed in rodents and have focused primarily on alterations to maternal diet (Chan *et al*. 2015b). It has been shown that accelerated ageing of the somatic ovarian tissue, with a concomitant decrease in ovarian reserve in early‐mid reproductive life, is a consequence of a maternal low protein diet (Aiken *et al*. 2013), an obesogenic maternal diet (Aiken *et al*. 2016) and maternal caloric restriction (Bernal *et al*. 2010) in various rodent models.

The primary outcome of most studies that have demonstrated a link between the early life environment and impairment of female fertility has been ovarian reserve (Chan *et al*. 2015b; Ho *et al*. 2017). As a key determinant of future reproductive potential (Depmann *et al*. 2015; Pelosi *et al*. 2015), ovarian reserve is a useful and specific marker of fertility potential, although reproduction depends on a wide range of factors beyond the availability of gametes. In the female, successful pregnancy depends not only on a viable oocyte, but also on a suitable reproductive tract environment. The oviduct has several vital roles in successful reproduction, including gamete transport (Wang & Larina, 2018), syngamy (Parada‐Bustamante *et al*. 2016) and early embryonic development (Robertson *et al*. 2015). Oviductal problems are a major cause of infertility in human populations, accounting for ∼25–35% of all female infertility (Practice Committee of the American Society for Reproductive, 2015). Such problems can range from complete blockage of the oviduct, which impairs gamete transport and prevents conception, to subclinical oviductal damage, such as via smoking, which alters the tubal epithelium and increases the risk of ectopic pregnancy (Horne *et al*. 2014; Nio‐Kobayashi *et al*. 2016). Impact on the oviductal environment of the adult offspring is thus an important consideration with respect to investigating the effect of developmental programming on female reproductive potential.

A limited number of studies have previously reported on the impact of an adverse intrauterine environment on the developing oviduct. Wister rat offspring exposed to a maternal low‐protein diet during gestation, followed by postnatal catch‐up growth, showed evidence of reduced telomere length and increased oxidative stress in the oviduct in early adulthood (Aiken *et al*. 2013). We hypothesize that exposure to chronic gestational hypoxia may also adversely affect the oviduct, and hence the peri‐conceptual environment, in a similar way.

Using an established model of hypoxic pregnancy in rats, we investigated the impact of exposure to a 40% reduction in environmental oxygen (13% *vs*. 21% ambient oxygen from day 6 of pregnancy) on the oviduct of the adult female offspring. A reduction in the environmental oxygen tension by 40% reflects the difference in oxygen availability between pregnancies occurring at sea level compared to 3500–4000 m a.s.l. (Postigo *et al*. 2009). Hence, our rat model of gestational hypoxia is highly relevant to human pregnancy at these altitudes, where it is estimated that ∼40,000 babies are born each year in Bolivia alone (Roost *et al*. 2009). The present study therefore aimed to evaluate whether there is evidence of accelerated ageing in the oviducts of young adult female rats exposed to chronic gestational hypoxia.

## Methods

### Ethical approval

All animal experiments were approved by the University of Cambridge Animal Welfare and Ethical Review Board (reference no. PC6CEFE59). All animal experiments were conducted in accordance with the British Animals (Scientific Procedures) Act (1986) and were compliant with EU Directive 2010/63/EU. Animals were killed by CO_2_ inhalation and cervical dislocation.

#### Study design

Wistar rat dams at 10–12 weeks of age (Charles River Ltd, Margate, UK; wild‐type RRID: RGD_13508588) were housed in individually ventilated cages (21% oxygen, 70–80 air changes h^–1^) under standard conditions, with a regular 12:12 h light/dark cycle. All animals were fed a standard laboratory chow diet (20% protein) and fed *ad libitum* with free access to water. After initial acclimatization (10 days), they were mated with fertile male Wistar rats, and pregnancy was confirmed via observation of a vaginal plug. The day of the plug was designated as day 0 of pregnancy (full term 21–22 days). Upon confirmation of pregnancy, dams were weighed and housed individually. On day 6 of pregnancy, dams were randomly divided into two groups: control (21%) and hypoxic (13%) pregnancy (*n* = 8 per group). Pregnant rats assigned to the hypoxia group were placed inside a chamber that could hold nine rat cages, which combined a polyvinyl chloride isolator with a nitrogen generator, as described previously (Giussani *et al*. 2012; Herrera *et al*. 2012). The hypoxia model did not alter maternal food intake or gestational length. Pregnancies undergoing hypoxia were maintained at a constant inspired fraction of oxygen of 13% from days 6 to 20 of gestation. All dams delivered under normoxic conditions, and normoxia (21%) was maintained for all animals during lactation, weaning and thereafter. Following determination of birth weight, litters were culled to four males and four females to standardize nutritional access and maternal care (Herrera *et al*. 2012). All pups were suckled by their own mothers. At 4 months of age, adult female pups were killed by CO_2_ inhalation and cervical dislocation. At postmortem, the reproductive tract tissues were harvested immediately after dissection. The oviducts were snap‐frozen in liquid nitrogen until used for analysis. No sample was refrozen after the initial thaw.

#### Telomere length analysis

High‐molecular weight DNA was extracted using the DNeasy Blood and Tissue kit (Qiagen, Hilden, Germany) in accordance with the manufacturer's instructions. DNA quantity and purity was determined using a Nanodrop spectrophotometer (Nanodrop Technologies, Wilmington, DE, USA). Agarose gels were run to ensure all DNA samples were of high‐molecular weight. DNA (1.2 μg) was digested with *Hinf*I and *Rsa*I restriction enzymes for 2 h at 37 °C. The restricted samples were quenched with 5 × SDS loading buffer (Roche Diagnostics, Mannheim, Germany) and loaded onto agarose gels containing SYBR safe stain (Invitrogen, Paisley, UK). After pulsed field gel electrophoresis, the gels were checked for non‐specific degradation of an undigested DNA control and complete digestion of the enzyme‐restricted DNA by visualizing the stained gels under UV light (Syngene, Cambridge, UK). The separated DNA fragments were transferred to nylon membrane (Roche Diagnostics) by Southern blotting, and telomeric repeat length was determined using a commercial method of chemiluminescent detection, as described previously (Tarry‐Adkins *et al*. 2006). Molecular weight markers on each gel were a mid‐range pulsed‐field gel marker (New England Biolabs, Ipswich, MA, USA) and dioxygenin (low range) molecular‐weight marker (Roche Diagnostics, Mannheim, Germany). Standard undigested and digested genomic samples of DNA from a 4 month control animal were also included on each gel to confirm digestion efficiency. Telomere signals were analysed using Photoshop (Adobe Systems Inc., San Jose, CA, USA) and Alpha Ease Software (Alpha Innotech, San Leandro, CA, USA). Telomere length was measured as described previously (Tarry‐Adkins *et al*. 2006).

#### Gene expression analysis

An initial panel of 38 candidate genes was developed to test which molecular pathways might be altered in the somatic oviduct following exposure to chronic gestational hypoxia. These genes were chosen based on (i) previous work on the effects of developmental programming on ovarian, para‐ovarian adipose tissue and oviductal gene expression (Aiken *et al*. 2015; Aiken *et al*. 2016; Tarry‐Adkins *et al*. 2018); (ii) knowledge of programming mechanisms in other organ systems in the same gestational hypoxia rat model (Camm *et al*. 2010; Giussani *et al*. 2012; Herrera *et al*. 2012); and (iii) a review of the relevant literature. RNA was extracted from snap‐frozen oviducts using a miRNeasy mini kit (Qiagen) in accordance with the manufacturer's instructions, with the addition of a *DNase*I digestion step to ensure no genomic DNA contamination. RNA quantification was performed using a NanoDrop spectrophotometer (Nanodrop Technologies). RNA (1 μg) was used to synthesize cDNA using oligo‐dT primers and M‐MLV reverse transcriptase (Promega, Madison, Wisconsin, USA). Gene expression was determined using custom designed primers (Sigma, Poole, Dorset, UK) and SYBR Green reagents (Applied Biosystems, Warrington, UK), as described previously (Tarry‐Adkins *et al*. 2009). Primer sequences are provided in the Supporting information (Table S1). Quantification of gene expression was performed using a Step One Plus RT‐PCR machine (Applied Biosystems). Equal efficiency of the reverse transcription of RNA from all groups was confirmed through quantification of expression of the house‐keeping gene *ppia*, for which the expression did not differ between groups.

#### Protein quantification

As a result of the extremely small amount of tissue available, limited protein quantification was performed. Genes were selected for protein expression analysis on the basis of (i) RNA quantification results and (ii) rationale from previous studies in the same model. Protein was extracted from whole tissue lysates of snap‐frozen oviducts, as described previously (Tarry‐Adkins *et al*. 2015; Tarry‐Adkins *et al*. 2018). Protein (20 μg) was loaded onto 10%, 12% or 15% polyacrylamide gels, depending upon the molecular weight of the protein to be measured. The samples were electrophoresed and transferred to polyvinylidene fluoride membranes. Detection steps used the following primary antibodies: P53 (R&D Systems, R&D Systems, Minneapolis, MN, USA; catalogue no. MAB1355; dilution 1:1000; RRID:AB_357649), P16^INK^ (Abcam, Cambridge, UK; catalogue no. Ab189034; dilution 1:1000; RRID:AB_2737282), OGG1 (Novus Biologicals, Littleton, CO, USA; catalogue no. NB100‐106; dilution 1:1000; RRID:AB_10104097), MRE11 (ProteinTech, Cambridge, UK; catalogue no. 10744‐1‐AP; dilution 1:1000; RRID:AB2145118), KU70 (ProteinTech; catalogue no. 10723‐1‐AP; dilution 1:1000; RRID:AB_), KU80 (Novus Biologicals; catalogue no. NB100‐508; dilution 1:1000; RRID:AB_2218756), Total Ox Phos rodent antibody cocktail (Abcam; catalogue no. Ab110413; dilution 1:5000; RRID:AB_2629281), HIF1α (Abcam; catalogue no. Ab51608; dilution 1:1000; RRID:AB_880418), GP91^phox^ (ProteinTech; catalogue no. 19013‐1‐AP; RRID:AB_1342287; dilution 1:1000), P47^phox^ (ProteinTech; catalogue no. 15551‐1‐AP; dilution 1:1000; RRID:AB_11182937), XO (Santa‐Cruz, Wimbledon, UK; catalogue no. SC‐20991; dilution 1:200, RRID:AB_2214858), HMOX1 (ProteinTech; catalogue no. 20960‐1‐AP; dilution 1:1000; RRID:AB_10732601), Catalase (Abcam; catalogue no. Ab1877‐10; dilution 1:10,000; RRID:AB_187710), MnSOD (Upstate, Watford, UK; catalogue no. 06‐984; RRID:AB_310325; dilution 1:1000), CuZnSOD (ProteinTech; catalogue no. 10269‐1‐AP; dilution 1:1000; RRID:AB_2193750). Anti‐rabbit secondary antibodies (Cell Signaling Technology, Danvers, MA, USA; dilution 1:2000) were utilized for all primary antibodies except P53, which required an anti‐mouse secondary antibody (Cell Signaling Technology; dilution 1:2000). Equal protein loading was confirmed by staining electrophoresed gels with Coomassie Blue (Bio‐Rad, Hemel Hempstead, UK) to visualize total protein. To ensure that the chemiluminescent signal changed in a linear manner, the ratio between loading controls (100% and 50% pooled sample) was confirmed for each detected protein.

#### Statistical analysis

All data were initially analysed using a two‐way ANOVA with gestational hypoxia/normoxia as the independent variable. Raw *P* values were transformed to take account of the false discovery rates. Maternal environmental effects were compared between groups using two‐tailed Student's *t* tests. Data are represented as the mean ± SEM. Where *P* values are reported, an alpha level <0.05 was considered statistically significant. All data analysis was conducted using the R, version 2.14.1 (R Foundation for Statistical Computing, Vienna, Austria). In all cases, *n* refers to the number of litters, with *n* = 7–8 for all groups. The adequacy of the sample size was determined via a power calculation based on the effect sizes for somatic ovarian expression for ageing‐related genes a previous rodent developmental programming model (Aiken *et al*. 2016) using an alpha level of 0.05 to give a power of 0.8. Sample analysis was performed using project codes to blind the investigators to the experimental groups.

## Results

There was no impact of chronic gestational hypoxia on either maternal food intake during gestation (normoxia 79 ± 2 g kg^–1^ day^−1^ *vs*. hypoxia 70 ± 3 g kg^–1^ day^−1^) or length of gestation (normoxia 20 ± 1 days *vs*. hypoxia 20 ± 1 days).

### Maintenance of oviductal telomere length

**Figure 1:**
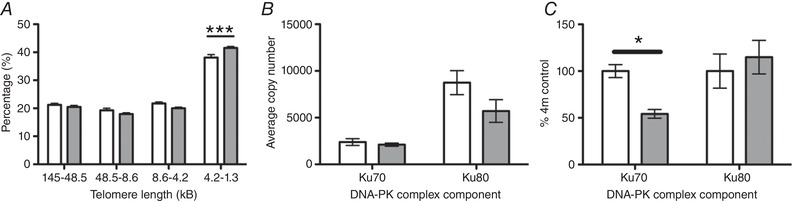
Oviductal telomere length (*A*), gene expression (*B*) and protein expression of DNA‐protein kinase complex components in oviducts (*C*). Control (open bars) v. hypoxia (grey bars) *A*, oviductal telomere length in adult female rats exposed to gestational hypoxia compared to normoxia. *B*, effect of gestational hypoxia compared to normoxia on gene expression of components (*Ku70* and *Ku80*) of the DNA‐activated protein kinase (DNA‐PK) in the oviducts. *C*, effect of gestational hypoxia compared to normoxia on protein expression of KU70 and KU80. Data shown as the mean ± SEM. Open bars: normoxia (21% oxygen) during gestation, grey bars: hypoxia (13% oxygen) during gestation. ^*^ *P* < 0.05, ^***^ *P* < 0.001. *n* = 7–8 for all groups (*n* refers to the number of litters)

At 4 months of age, there were significantly more very short (1.3–4.2 kB, *P* < 0.001) telomeres in the oviducts of gestational hypoxia‐exposed adult females compared to the normoxic group (Fig. 1 *A*). There were no significant differences between the hypoxia and normoxia‐exposed groups in the proportion of telomeres that were short (4.2–8.6 kB), long (8.6–45.5 kB) or very long (45.5–145 kB).

### Cell‐cycle markers of ageing

Alongside the increase in very short telomeres observed in hypoxia‐exposed tissues, there was an increase in cell‐cycle markers that increase with cellular ageing. Gene expression of *p21* was significantly increased in the hypoxia‐exposed group compared to the controls (*P* < 0.04). There was also a trend towards increased *p53* expression (*P* = 0.09), although this did not reach statistical significance (Table 1). At the protein expression level, there was no significant difference in P16ink levels between groups, although there was a significant increase in P53 (*P* < 0.05) (Table 2).

**Table 1:** Effect of gestational hypoxia compared to normoxia on gene expression in the oviducts of adult female rats

Gene	Normoxia	Hypoxia	
*Ppia*	32234 ± 2363	28269 ± 3394	NS
*P53*	10775 ± 1237	13417 ± 1332	0.09
*P21*	5188 ± 1053	9292 ± 1374	0.04
*Alox12*	3120 ± 744	7714 ± 2089	0.05
*Alox15*	925 ± 225	854 ± 147	NS
*Ogg1*	1294 ± 135	1710 ± 132	0.03
*Neil1*	769± 63	730 ± 117	NS
*Nth1*	1505 ± 27	1329 ± 151	NS
*Xrrc1*	2675 ± 375	2175 ± 372	NS
*Nrf2*	11560 ± 1704	7555 ± 893	NS
*Dna pkcs*	2134 ± 323	1421 ± 192	0.1
*Mre11*	723 ± 119	307 ± 79	0.04
*Ku70*	2380 ± 397	1533 ± 389	NS
*Ku80*	8743 ± 1410	5709 ± 1219	NS
*Bax*	2093 ± 199	1750 ± 329	NS
*Bcl2*	4036 ± 530	2599 ± 293	0.05
*BaxBcl2*	0.41 ± 0.02	0.5 ± 0.08	NS
*Tfam*	6447 ± 844	3866 ± 632	0.04
*Pgc1a*	1806 ± 121	903 ± 236	0.01
*Cs*	18621 ± 2551	9627 ± 156	0.02
*Lonp1*	7518 ± 874	7262 ± 1035	NS
*Cycs*	27321 ± 4613	15812 ± 4446	0.08
*Complex I*	26745 ± 721	22123 ± 2086	0.01
*Complex II*	19112 ± 3730	14311 ± 1389	NS
*Complex III*	27555 ± 4854	18414 ± 1721	NS
*Complex IV*	46402 ± 4883	33668 ± 1533	0.05
*Hif*	8172 ± 791	8276 ± 628	NS
*Gp91phox*	6191 ± 1727	6904 ± 1023	NS
*P22phox*	5128 ± 1081	7298 ± 1030	NS
*P47phox*	1887 ± 136	2620 ± 631	NS
*Xo*	19493 ± 2381	15989 ± 1793	NS
*Gpx1*	67342 ± 11501	34576 ± 8409	NS
*Hmox1*	3492 ± 202	3720 ± 255	NS
*Catalase*	12593 ± 1716	13651 ± 280	NS
*Nfkβ*	6419 ± 476	6073 ± 307	NS
*Mnsod*	9286 ± 2005	15399 ± 577	0.04
*Cuznsod*	171954 ± 8398	160528 ± 13018	NS
*Ecsod*	35354 ± 3730	23778 ± 3163	NS

*n* = 7–8 for all groups (*n* refers to the number of litters). NS, not significant.

All reported *P* values have been adjusted to take account of multiple hypothesis testing.

**Table 2:** Effect of gestational hypoxia compared to normoxia on protein expression in the oviducts of adult female rats

Protein	Normoxia	Hypoxia	
P53	100 ± 17	158 ± 19	0.05*
P16^INK^	100 ± 30	100 ± 24	NS
OGG1	100 ± 22	137 ± 13	0.08
MRE11	100 ± 30	77 ± 22	NS
KU70	100 ± 10	58 ± 12	0.03*
KU80	100 ± 18	115 ± 18	NS
Complex I	100 ± 36	142 ± 56	NS
Complex II	100 ± 29	150 ± 38	NS
Complex III	100 ± 15	96 ± 18	NS
Complex IV	100 ± 22	137 ± 31	NS
Complex V	100 ± 2	108 ± 6	NS
CS	100 ± 13	110 ± 16	NS
HIF1α	100 ± 12	124 ± 15	NS
GP91^phox^	100 ± 27	97 ± 15	NS
P47^phox^	100 ± 24	119 ± 4	NS
XO	100 ± 10	92 ± 11	NS
HMOX1	100 ± 44	37 ± 11	NS
CATALASE	100 ± 10	125 ± 23	NS
MnSOD	100 ± 9	156 ± 10	<0.01**
CuZnSOD	100 ± 30	94 ± 23	NS

^*^ *P* < 0.05, ^**^ *P* < 0.01. *n* = 7–8 for all groups (n refers to the number of litters). NS, not significant.

All reported *P* values have been adjusted to take account of multiple hypothesis testing.

### DNA damage repair mechanisms

Gene expression of *Ogg1* was elevated in the hypoxia‐exposed group compared to the normoxic group (1294 ± 135 *vs*. 1710 ± 132 units; *P* < 0.05) (Table 1). At the protein level, the elevation of OGG1 in the hypoxia‐exposed group was of borderline significance (*P* = 0.08) (Table 2). By contrast *Mre11* expression was decreased by more than 50% in the hypoxia‐exposed group compared to the controls (723 ± 119 *vs*. 307 ± 79, *P* < 0.05) (Table 1); however, there was no difference in MRE11 protein expression between the experimental groups (Table 2). There was a trend towards an overall reduction in the catalytic subunit of the DNA protein kinase (*DNA pkcs)* that is required for double‐stranded break repair and telomere maintenance (*P* < 0.1) (Table 1), although there were no differences in the expression of either of the components of the binding subunit, *Ku70 or Ku80* (Fig. 1 *B*). However, at the protein level, there was a significant deficit of KU70 in the oviducts of animals exposed to gestational hypoxia (*P* < 0.05), with no difference in KU80 levels (Fig. 1 *C*).

There was no significant difference between hypoxia‐exposed and normoxic groups in expression of any other DNA damage sensing or early repair mechanisms that were included in the candidate genetic screen: *Neil1*, *Nthl1* or *Xrcc1* (Table 1).

### Mitochondrial biogenesis

**Figure 2:**
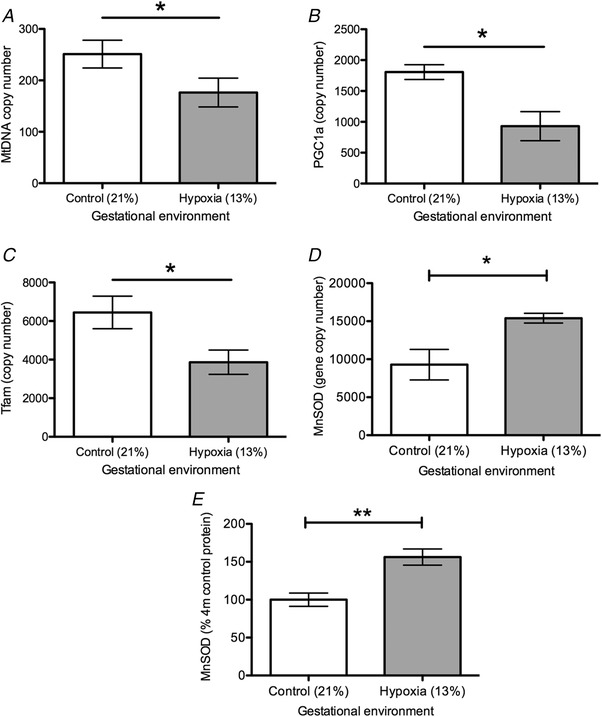
Effect of gestational hypoxia on mitochondrial parameters Effect of gestational hypoxia compared to normoxia on expression of mitochondrial biogenesis regulators and mitochondrial anti‐oxidant defence in the oviducts. Data are shown as the mean ± SEM. Open bars: normoxia (21% oxygen) during gestation, grey bars: hypoxia (13% oxygen) during gestation. *A*, mtDNA copy number. *B*, *Tfam* gene expression. *C*, *Pgc1α* gene expression. *D*, *MnSOD* gene expression. *E*, MnSOD protein expression. ^*^ *P* < 0.05, ^**^ *P* < 0.01. *n* = 7–8 for all groups (*n* refers to the number of litters)

mtDNA copy number was reduced in hypoxia‐exposed animals compared to controls (*P* < 0.05) (Fig. 2 *A*). The expression of *Tfam* was significantly reduced in oviducts of animals exposed to gestational hypoxia compared to normoxic controls (*P* < 0.05) (Fig. 2 *B*). *Pgc1α* also showed reduced expression in the hypoxia‐exposed group (*P* < 0.05) (Fig. 2 *C*). There was no difference between groups in expression of *Nrf2* or *Lonp1* (Table 1). Hence, there is evidence that mtDNA biogenesis may be impaired in the oviduct after exposure to chronic gestational hypoxia.

We further investigated the gene expression of components of the mitochondrial respiratory complex. There was significant reduction in gene expression of complex I (*P* < 0.01) and complex IV (*P* < 0.05) in the hypoxia‐exposed group compared to the normoxia group. There was also a significant reduction in the gene expression of citrate synthase (*Cs*) (*P* < 0.05) (Table 1). There was no difference in the expression levels of complex II, complex III or cytochrome C (*Cycs*). However, there was no significant difference in protein expression between the hypoxia‐exposed and normoxia‐exposed groups in any of the tested mitochondrial respiratory components (Table 2).

### Oxidative stress and anti‐oxidant defence capacity

There was no direct evidence of increased oxidative stress markers in any of the pathways tested in the oviducts at either the gene expression or protein levels (*Hif1α*, *Gp91phox*, *P22phox*, *P47phox*, *Xo*, *Gpx1* and *Hmox1)* (Tables 1 and 2).

In terms of anti‐oxidant defence capacity, there was no significant difference in gene expression of *Catalase*, *Cuzusod* or *Ecsod* in the hypoxia‐exposed compared to the normoxia group. However, there was an increase in *MnSOD* expression at both the RNA and protein level (Fig. 2 *D* and *E*), which is in keeping with the suggestion that mitochondrial biogenesis may be suboptimal in the gestational oviduct. MnSOD is the specific mitochondrial isoform of the powerful superoxide dismutase group of anti‐oxidants. Increased expression of MnSOD may thus indicate a successful attempt to buffer the impact of excess free radical generation resulting from impaired mitochondrial biogenesis.

### Lipid peroxidation

There was a significant increase in the gene expression of *Alox12* (a key component of the lipoxygenase pathway) in the hypoxia‐exposed group compared to the controls (*P* < 0.05) (Table 2). There was no difference in the gene expression levels of *Alox15* between the hypoxia‐exposed and control groups.

## Discussion

In the present study, we provide evidence of accelerated ageing in the oviducts of female offspring in early‐mid adulthood, following exposure to chronic gestational hypoxia. Accelerated ageing is demonstrated at the cellular level by decreased telomere length and increased expression of markers of cellular ageing, in particular *p21* and *p53*. The observed decrease in oviductal telomere length was accompanied by a specific post‐transcriptional reduction in KU70, which is a key functional subunit of the DNA‐activated protein kinase required for telomere length maintenance (Jette & Lees‐Miller, 2015). The observed upregulation of *Ogg1* in the oviducts of the hypoxia‐exposed animals is in keeping with an increase in oxidative DNA damage. *Ogg1* excises 7,8‐dihydro‐8‐oxoguanine from damaged DNA, which limits the impact of ubiquitous oxidative damage accumulated during normal ageing (Radicella *et al*. 1997). Hence, the observed increase in *Ogg1* suggests a greater exposure to oxidative DNA damage in the oviducts following gestational hypoxia.

Clear evidence was also provided indicating that mitochondrial biogenesis is reduced in the oviduct following exposure to chronic gestational hypoxia. In particular, the key regulatory genes controlling mitochondrial biogenesis (*Tfam* and *Pgc1α*) were both downregulated in the hypoxia‐exposed group compared to the controls. *Tfam* is the master regulator of mitochondrial biogenesis via gene expression from the mitochondrial genome (Picca & Lezza, 2015) and *Pgc1α* regulates mitochondrial biogenesis via nuclear gene expression (Picca & Lezza, 2015). Alongside the observed reduction in mtDNA copy number, there is thus evidence that both key mechanisms regulating mitochondrial biogenesis are impaired following exposure to gestational hypoxia. Evidence of a mitochondrial deficit is particularly interesting because oviductal function depends on ciliary motility and co‐ordinated smooth muscle contraction (Halbert *et al*. 1976; Bylander *et al*. 2013; Zhao *et al*. 2015). Both of these processes are dependent on normal mitochondrial function and ATP production (Dirksen & Zeira, 1981; Lydrup & Hellstrand, 1986), particularly in the ciliated cells of the oviduct epithelium. Oviductal ultra‐structure, including mitochondria in the ciliated epithelial cells, appears to be established mainly during late fetal life (Kenngott *et al*. 2008; Zhao *et al*. 2015), which correlates with the timing of exposure to a chronic hypoxic environment in the present study.

There is remarkably little published evidence regarding oviductal phenotype in other developmental programming models, despite the plethora of studies that have examined ovarian reserve (Bernal *et al*. 2010; Aiken *et al*. 2013; Chan *et al*. 2015a; Aiken *et al*. 2016). However, at least one previous study has examined the impact of a maternal low protein diet on mtDNA copy number and telomere length in the oviduct (Aiken *et al*. 2013). In keeping with the findings of the present study, oviductal telomere length was shown to be particularly sensitive to the early life environment, more so than the somatic ovarian tissue (Aiken *et al*. 2013), and this effect was magnified with increasing age (Aiken *et al*. 2013). In the present study, we observed the same highly significant reduction in telomere length in young animals near the start of reproductive life. An important point for future development of this work is to test directly whether oviductal shortening in response to gestational hypoxia is magnified later in reproductive life. Interestingly, in response to a maternal low protein diet, oviductal mtDNA copy number was increased compared to the controls (Aiken *et al*. 2013), which contrasts with the findings of the present study. This suggests that reduced mitochondrial biogenesis is a specific effect of gestational hypoxia rather than a generic impact of early life stress on the oviduct. The relatively small number of proteins in the developing oviduct affected by exposure to gestational hypoxia also points towards a highly specific impact on cellular ageing within the oviduct, rather than ubiquitous tissue damage caused by the adverse early life environment. We also did not observe ubiquitous upregulation of markers of oxidative stress in the oviducts (*Hif1α*, *Gp91phox*, *P22phox*, *P47phox*, *Xo*, *Gpx1* and *Hmox1)*, which are normally highly sensitive to generic tissue damage adding further evidence that the effect reported is highly specific.

In keeping with the strong evidence of reduced mitochondrial biogenesis in the hypoxia‐exposed oviducts, we also observed an increase in mitochondrial‐specific anti‐oxidant defence. MnSOD was upregulated in the hypoxia‐exposed group compared to the controls, indicating that there may be an increase in reactive oxygen species produced. Mitochondria are the major intracellular source of reactive oxygen species, although there was no direct evidence of an increase in any of the oxidative stress markers that were assayed in this study. However this may become apparent as the animals age.

Oviducts are a relatively homogeneous tissue, with very low levels of telomerase expression (Lee *et al*. 2001). This is a significant advantage for the present study, which provides novel insight into this relatively under‐studied yet crucial part of the female reproductive system. A limitation of the present study is the inherently tiny amount of tissue available from each experimental animal (average oviductal weight ≤5 mg) (Sen & Talwar, 1973). This meant that the assays performed on protein, RNA and DNA had to be strictly prioritised rather than testing all potential genes and proteins of interest. The extremely small mass of the tissue also meant that we were unable assign tissue for histological examination or cell‐type specific analysis. These are important aims for future work. In particular, future studies should focus on whether the muscularis or the epithelium or both are affected by the phenotype described. Either could plausibly have a significant influence on oviductal function and future fertility. Accelerated ageing in the muscularis could affect efficient transport of gametes or conceptus, thus influencing the future risk of ectopic pregnancy. Accelerated ageing in the epithelium could influence the composition of the oviductal fluid, and hence the culture medium for the early embryo. Assessing oviductal function *in vivo*, including assessing fertility outcomes, would help to confirm the implications of our results and refine our understanding of the phenotype. All these considerations should form the basis of future investigations.

Oviduct‐related infertility is a key cause of female subfertility, accounting for ∼30% of cases (Kawwass *et al*. 2013), and increases with advancing maternal age (Maheshwari *et al*. 2008). Our work suggests that there may be a developmentally programmed component to the acceleration in cellular ageing and hence oviductal dysfunction observed in women aged ≥35 years (Maheshwari *et al*. 2008). The age of the animals investigated in the present study equates to early in reproductive life, and hence the observed evidence of cellular ageing in the oviducts is even more striking. Aside from infertility, the ageing of the oviducts is a significant risk factor predisposing to tubal ectopic pregnancy (Nybo Andersen *et al*. 2000), which can be a fatal complication of oviductal dysfunction (Farquhar, 2005). The risk of ectopic pregnancy increases sharply with maternal age from 1.4% of all pregnancies in women aged 21 years to 6.9% of pregnancies in women aged >44 years (Nybo Andersen *et al*. 2000). The active role of the oviductal epithelium in the pathogenesis of ectopic pregnancy is becoming increasingly clear (Horne & Critchley, 2012), as is the requirement for normal regulation of smooth muscle contractility (Shaw *et al*. 2010). Hence, our finding that adult females who have been exposed to chronic gestational hypoxia show accelerated ageing and dysregulated mitochondrial biogenesis in the oviducts may have potential clinical significance not only for patients with difficulty conceiving but also in understanding risk factors for ectopic pregnancy.

## Conclusions

Large numbers of pregnancies worldwide are exposed to chronic gestational hypoxia, either through pregnancy at high altitude or through utero‐placental insufficiency (Ducsay, 1998; Kuzmina *et al*. 2005; Postigo *et al*. 2009; Giussani *et al*. 2016). The recognition of the adverse impact of lower than normal oxygenation during pregnancy on ageing of the oviducts, with attendant consequences for gamete and embryo transport in potential next generation mothers, is an important area for further research and exploration.

## Additional information

### Competing interests

The authors declare that they have no competing interests.

### Author contributions

CEA, DAG and SEO conceived the study. DAG and SEO designed the study. JTA, AMS, AMN, TJA, AR, MJS and EJC acquired the data. CEA, JTA, AMS, AMN, TJA, AR, MJS and EJC analysed the data. CEA interpreted the data. CEA, DAG and SEO drafted the manuscript. JTA, AMS, AMN, TJA, AR, MJS and EJC critically revised the manuscript. All of the authors approved the final version of the manuscript. All authors agree to be accountable for all aspects of the work in ensuring that questions related to the accuracy or integrity of any part of the work are appropriately investigated and resolved. All persons designated as authors qualify for authorship, and all those who qualify for authorship are listed.

### Funding

CEA was supported by a grant from the Addenbrooke's Charitable Trust (ACT; RG94137) and by an Issac Newton Trust/Wellcome Trust ISSF/ University of Cambridge Joint Research Grant. SEO is supported by the MRC (MC_UU_12012/4). DAG is supported by The British Heart Foundation (PG/14/5/30546).

## Supporting information


**Table S1**: Primer sequences and product length for reported genes
