# Supplementary material for: Chronic fetal hypoxia disrupts the peri‐conceptual environment in next‐generation adult female rats
Source: J Physiol. 2019 Mar 24;597(9):2391–401. doi: 10.1113/JP277431 (PMC6487938; doi:10.1113/JP277431)
Supplement: Supplementary file 1 — Table S1: Primer sequences and product length for reported genes [file TJP-597-2391-s001.doc]

| **Primer** | **Sequence (F)** | **Sequence (R)** | **Product size (bp)** |
| --- | --- | --- | --- |
| ***Ogg1*** | CTTAATGGCCCTGGACAAAC | CGTAGTCACGATGGGCAAT | 74 |
| ***Nth1*** | GATTTTGCCTTCCTGTCCATC | GAAGCCCAAAACCCTCAGA | 79 |
| ***Neil1*** | CAGAAGGTCAAGGCCAAACT | CTCACCCAGCTGAACCACTT | 84 |
| ***Xrcc1*** | GGGGAGAAGGCACAGAGC | TCTGGAAGCCACCTGAGCAC | 96 |
| ***Ku70*** | ACTGAGGGACATCTGCAAGG | ACTGAGGGACATCTGCAAGG | 84 |
| ***Ku80*** | GACATGAAGCTCTGGCCATC | TGTCTGTAGGGACCTGGAGTG | 68 |
| ***P53*** | CCTATCCGGTCAGTTGTTGG | CGTATGAGGGCCCAAGATAG | 89 |
| ***P21*** | TGCAAGAGAAAGCCCTGAAG | TGAATGAAGGCTAAGGCAGAA | 96 |
| ***Hif1α*** | TCAAAAGCAGTGACGAAGGA | TGGGTAGAAGGTGGAGATGC | 68 |
| ***Nfκβ*** | CTTCTCGGAGTCCCTCACTG | TAGGTCCATCCTGCCCATAA | 80 |
| ***Xo*** | GAGAAGGTCTCCAGCAGTGG | GCATGCGGAAATCTGGATA | 86 |
| ***Gp91phox*** | CGAAGCCTTGGCTAAAACTCT | TCCTTGTTGAAGATGAAGTGGA | 87 |
| ***P22phox*** | GTGAGCAGTGGACTCCCATT | GTAGGTGGCTGCTTGATGGT | 76 |
| ***P47phox*** | CCGATAACCGGACAACAGAG | CAGGTCTTCTGGCTGGGTAG | 72 |
| ***Nrf2*** | AGCAAGACTTGGGCCACTTA | GATGGAGGTTTCTGTCGTTTTC | 78 |
| ***Hmox1*** | TAACCAGGATCTCCCCAAGA | TTAGAGTGCTGTGGCAGGTG | 71 |
| ***Gpx1*** | CACCCGCTCTTTACCTTCCT | CGGGGACCAAATGATGTACT | 75 |
| ***Mnsod*** | TGACTATGTAATGTTTTATCAGTTGGA | GTTGCTGACCACAGCCTTTT | 91 |
| ***Cuznsod*** | TTGTGGTGTGATTGGGATTG | CAGTTTAGCAGGACAGCAGATG | 80 |
| ***Ecsod*** | ATCCCATAAGCCCCTAGCAT | ATTCGACCTCTGGGGGTAAG | 84 |
| ***Catalase*** | TTGGATCATGTCTTCCAAAAA | GGGAAAAGGAATCCGATCAA | 83 |
| ***Alox12*** | TGTGCTCAGCCAATTTCAAG | GGTATTCGTAGGGCAGGTCA | 89 |
| ***Alox15*** | GCTGTGCTGAAGAAGTTCAGAG | GCCGCAGGT ACT CAT AAG GT | 85 |
| ***Ppia*** | TGAGAACTTCATCCTGAAGCATACA | CATTTGTGTTTGGTCCAGCATT | 89 |
| ***Dna pkcs*** | CAACATTCGTGCACAAGAGC | CTGATCCACCAAGCACTTCA | 70 |
| ***Mre11*** | CTCAGCCTTCAGTGCAGATG | CACTGCTGACTGGCTATCGT | 82 |
| ***Bax*** | TGACATTTTTCCTGGGATG | CCACCCCCTCCCAATAATTACA | 74 |
| ***Bcl2*** | TGTCTCTGAAGACGCTGCTC | CTCACTTGTGGCCCAGGTAT | 78 |
| ***Tfam*** | GTCTTGGGAAGAGCAAATGG | TTCACACTGCGACGGATG | 74 |
| ***Pgc1a*** | TTA CAC CTG TGA CGC TTT CG | TTG CTT CCG TCC ACA AAA GT | 84 |
| ***Cs*** | TCCTGTTCGGAGTGTCGAG | CATGGACTTGGGCCTTTCTA | 92 |
| ***Lonp1*** | TCTCGACTTGGCTCCCTTC | GGAAGATGTCGCGGTAGTGT | 72 |
| ***Cycs*** | GGGAGAGGATACCCTGATGG | TGCCCTTTCTCCCTTCTTCT | 100 |
| ***Complex I*** | TGGCAAGAAAAATGTTGCAG | TATTGGCCACTTCCACTGGT | 80 |
| ***Complex II*** | GGATCAGATTGTGCCTGGTC | TCCAAAAGAGAGTTTGCTCCA | 93 |
| ***Complex III*** | AAGCTTTGCCAGAGTTTCCA | ATGCTCATGGCATCACAGAC | 89 |
| ***Complex IV*** | TCCCTCATACCTTTGATCGTG | GTTGACCTTCATGTCCAGCA | 68 |
